# Genetic variants in VWF exon 26 and their implications for type 1 Von Willebrand disease in a Saudi Arabian population

**DOI:** 10.1515/biol-2025-1087

**Published:** 2025-11-17

**Authors:** Faisal M. Alzahrani, Asma A. Al Faris, Layla A. Bashawri, Maryam A. Aldossary, Osama Al Sultan, Ahmed Mousa Alzahrani, Thekra N. Al-Maqati, Elmoeiz A. Elnagi, Fathelrahman Mahdi Hassan

**Affiliations:** Department of Clinical Laboratory Sciences, College of Applied Medical Sciences, Imam Abdulrahman Bin Faisal University, Dammam, Saudi Arabia; Department of Internal Medicine, King Fahad Hospital of the University, Imam Abdulrahman Bin Faisal University, Khobar, Saudi Arabia; Blood Bank, Department of Pathology and Laboratory Medicine, King Abdulaziz Medical City in Jeddah, Ministry of National Guard, Jeddah, Saudi Arabia; Department of Clinical Laboratory Sciences, Prince Sultan Military College of Health Sciences, Dhahran, Saudi Arabia; Department of Hematology and Immunohematology, College of Medical Laboratory Science, Sudan University of Science and Technology, Khartoum, Sudan

**Keywords:** exon 26, sanger sequencing, Von Willebrand Disease type 1, Von Willebrand factor

## Abstract

Von Willebrand disease (VWD) is one of the most common bleeding disorders, stemming from irregularities in the Von Willebrand factor (VWF). Globally, type 1 VWD (VWD1) is the most prevalent form, characterized by decreased levels of VWF in the blood. Genotyping studies have found causative genetic variants in approximately 65% of VWD1 cases, revealing the presence of ethnic-specific VWF variants. This study is limited by its exclusive focus on exon 26, warranting further investigation of other exons and the inclusion of functional analyses. Moreover, these findings emphasize the importance of understanding genetic variability in population-specific contexts, supporting the necessity for future studies on additional exons and potential epigenetic interactions. Between 2018 and 2019, samples and relevant medical data were collected at King Fahad Hospital of the University in Al Khobar. Sanger sequencing of the exonic and flanking intronic regions of exon 26 was performed on 22 index cases clinically diagnosed with VWD1 and their first-degree relatives. Analysis revealed four exonic and 11 intronic variants in VWF exon 26 among the participants. None of the identified variants appeared to explain the VWD-related clinical features in these individuals. However, findings suggest a higher prevalence of specific VWF variants within the Saudi population compared to global databases. Further investigation, including sequencing of additional exons or next-generation sequencing, may help to uncover potential disease-causing variants in this cohort. Establishing a national sequencing project could also enhance our understanding of both common and rare variants in the genetic basis of complex diseases, such as VWD1.

## 1 Introduction

Von Willebrand Disease (VWD) was first identified in 1962 by Eric Von Willebrand in families experiencing excessive mucosal bleeding [1]. Today, VWD is known as the most common hereditary bleeding disorder, arising from either qualitative or quantitative abnormalities in the Von Willebrand factor (VWF) protein, which is encoded by the VWF gene [2]. Located on chromosome 12 on the reverse strand, the VWF gene spans approximately 178 kb [3]. The initial VWF transcript is 8.8 kb long, containing 52 coding exons, and produces a precursor VWF protein (pre-pro-VWF) of 2,813 amino acids and a mass of 250 kDa [4]. This precursor undergoes extensive post-translational modifications to form the mature VWF glycoprotein, which ranges from 500 to 10,000 kDa in molecular size [5]. The bleeding tendencies observed in VWD reflect VWF’s vital role in hemostasis, where it promotes platelet adhesion to areas of blood vessel damage by linking subendothelial collagen with platelet glycoprotein Ibα, and also serves as a carrier for coagulation factor VIII (FVIII) [6]. VWD is categorized into three primary types based on the specific nature of VWF abnormalities: Partial quantitative deficiencies are associated with type 1, complete quantitative deficiencies with type 3, and qualitative deficiencies with type 2 [7]. Among these, type 1 VWD (VWD1) is the most common and is typically associated with reduced VWF levels, generally between 5 and 50 IU/dL [8,9].

The level of VWF in plasma is known to be influenced by various genetic and environmental factors, complicating the diagnosis of VWD, particularly type 1 [10]. Although certain inherited factors, such as genetic variants in the VWF gene and the ABO blood group, are well-documented determinants of plasma VWF levels [11], other contributing factors remain less established. Research estimates that variability in the VWF gene accounts for approximately 5% of the variation, with the ABO blood group locus contributing about 25%, while additional genetic and environmental factors contribute roughly 35% each to the overall variability in VWF plasma levels [12,13]. Ethnicity has also been shown to influence VWF levels, with population genomic studies highlighting that the VWF gene exhibits high polymorphism in both its intronic and exonic regions, resulting in multiple ethnic-specific variants [12]. For instance, studies on healthy African-American populations identified common VWF variants that had previously been considered rare disease-causing mutations in European populations [14]. Due to this multifactorial complexity, identifying the genetic basis of VWD1 in patients is challenging [15]. Large genetic studies have successfully pinpointed causative variants in the VWF gene for only around 65% of VWD1 cases, with the underlying mechanisms remaining unknown for the remaining cases [16]. Despite advances in molecular diagnostics and a deeper understanding of VWF biology, the genetic diversity within the VWF gene and the disease’s variability make it difficult to identify the causative variants, which are scattered throughout the VWF gene and often show low penetrance [17]. Few studies have examined VWD1 within the Saudi population, focusing primarily on the disease’s prevalence and clinical characteristics [18]. The study adheres to the latest classification and VWF: Ag thresholds outlined in the ASH ISTH NHF WFH 2021 guidelines [19]. The aim of this study is to establish a pilot phenotypic-genotypic association study on Saudi patients clinically diagnosed with VWD1. Following the collection of phenotypic data, Sanger sequencing was employed to examine specific exons known to contain disease-causing variants, according to the Sheffield University VWF Online Database (http://www.vwf.group.shef.ac.uk/). This study focuses on exon 26 and its flanking intronic regions to identify any potential VWD1-associated variants [20]. Exon 26 was selected due to its known significance in global databases for harboring VWF variants. Unlike prior studies in Saudi Arabia that focused on exons such as 18 and 28, this study aims to provide novel insights by examining exon 26, an underexplored region in this population.

## 2 Methods

This research project is supported by the National Science, Technology and Innovation Program (NSTIP) under the King Abdulaziz City for Science and Technology (KACST) in Saudi Arabia, with the award number (11-MED1417-46). The comprehensive details of the participants and methods employed in this study have been previously documented [21]. In brief, this case-control study encompasses 22 index cases (ICs) diagnosed clinically with VWD1, alongside their first-degree relatives. These relatives were categorized into affected family members (AFM), consisting of 21 individuals, and unaffected family members (UAFM), comprising 17 individuals. This brings the total number of participants to 60. Prior to participation, informed consent was secured from all individuals or their legal guardians, ensuring ethical standards were upheld throughout the study. The phenotypic data collected from each participant included a variety of clinical parameters. This encompassed the International Society on Thrombosis and Hemostasis (ISTH) bleeding score, which quantifies bleeding severity and frequency.

### 2.1 Laboratory analysis

A range of coagulation assessments were performed, including the activated Partial Thromboplastin Time, which measures the time taken for blood to clot, and the Ristocetin cofactor activity (VWF:RCo), which evaluates the functional activity of VWF [22]. The levels of VWF antigen (VWF:Ag) were determined, as well as FVIII activity, which assesses the activity of coagulation factor VIII. Other relevant parameters included platelet count and the determination of ABO blood type, which is known to influence VWF levels.


**Informed consent:** Informed consent has been obtained from all individuals included in this study.
**Ethical approval:** The research related to human use has been complied with all the relevant national regulations, institutional policies and in accordance with the tenets of the Helsinki Declaration, and has been approved by the institutional review board at Imam Abdulrahman Bin Faisal University (IRB-2017-03-009).

### 2.2 Genetic analysis

DNA extraction was carried out using the ReliaPrep™ Blood gDNA MiniPrep System Kit (Promega Corporation, USA) from EDTA-preserved blood samples. The extraction process involved the lysis of cells, removal of proteins, and purification of DNA, yielding high-quality genomic DNA suitable for subsequent analysis. The study data and biological samples were meticulously gathered between 2018 and 2019 from King Fahad Hospital of the University in Al Khobar, ensuring a standardized approach to participant recruitment and data collection. This setting provided access to a diverse patient population, enhancing the representativeness of the findings. DNA quality was assessed using A260/A280 spectrophotometric ratios, with optimal values between 1.8 and 2.0. Agarose gel electrophoresis verified DNA integrity, confirming high-molecular-weight and intact DNA for analysis. Amplification of exon 26 was performed using forward (5′-CAGTGACTCATACCTGTAATCC-3′) and reverse primers (5′-GCTGTACCATAGGATCGTGAC-3′), which contain a mismatch to ensure specific amplification of VWF rather than the VWF pseudogene. These primers, previously used in related studies [23], were supplied by Invitrogen (Thermo Fisher Scientific™, USA). The target PCR product is 864 base pairs (bp), covering exon 26 (159 bp), the terminal portion of intron 25 (314 bp), and the initial section of intron 26 (391 bp) [24]. PCR reagents were sourced from MOLEQULE-ON^®^ Company, New Zealand, and reactions were carried out on a Maxi™ thermal cycler from ESCO Technologies, USA. PCR was set up using 100–200 ng of genomic DNA, 10 µM of each primer, and 15 µL of 1.1X ReddyMix PCR master mix in a total volume of 30 µL. The cycling conditions included an initial denaturation at 94°C for 7 min, followed by 32 cycles of denaturation at 94°C for 60 s, annealing at 60°C for 60 s, and extension at 72°C for 1 min, with a final extension step at 72°C for 7 min. Agarose gel electrophoresis on a 2% gel was then used to confirm the size of the PCR product before sequencing. Strict protocols minimized contamination, including using negative controls, aerosol-barrier pipette tips, and physical separation of workspaces. Regular decontamination of equipment ensured sample purity during PCR setup and sequencing workflows. Sequencing accuracy was ensured through repeated analysis of 10% of samples, bidirectional sequencing, and alignment with reference sequences. Manual inspection resolved ambiguities, verifying the reliability of the sequencing data. The desired band was extracted and purified using the MQ PCR/Gel product purification kit from MOLEQULE-ON^®^, New Zealand. Sanger sequencing was performed on the purified product with the Applied Biosystems ABI 3730xl Capillary DNA Analyzer (Applied Biosystems) using both forward and reverse primers. The resulting sequence data were compared with the reference human VWF sequence (NM 000552.4 and NC_000012.12 region: 5949015-6123196) as outlined in previous protocols [24]. Variant information was obtained through the Ensembl genome browser (GRCh37 Release 109) and the Ensembl Variant Effect Predictor (VEP) tool [25].

## 3 Results

Sanger sequencing of VWF exon 26 identified a total of 4 exonic and 11 intronic variants in 17 individuals (28%), of whom 11 were affected cases (including both ICs and AFM). Table 1 presents the demographic characteristics and laboratory parameters for subjects with identified variants in VWF exon 26. Table 2 shows that no significant association was observed in exonic variants of VWF exon 26 in VWD cohort (%) compared to control group (*p* = 0.565). The majority of subjects with exon 26 variants were female (15 participants, 88%) and had blood group O (15 participants, 88%). Their median age was 28 years (ranging from 8 to 60 years), and their median bleeding score was 2 (range 0–10). The mean VWF antigen (VWF:Ag) level was 66 U/dL, VWF:RCo was 53 U/dL, and FVIII activity was 61 U/dL. Table 3 details the pathogenicity of identified variants. Analysis of all detected variants revealed that eight were classified as variants of uncertain significance. Among these, a novel missense mutation was identified in the VWF gene: c.3346A > G (p.Asn1116Ser). This substitution affects a conserved amino acid within the D’ domain of VWF, as analyzed through bioinformatics tools such as SIFT and PolyPhen-2. Pathogenicity was assessed using SIFT, PolyPhen-2, and SpliceAI, providing functional predictions for both exonic and intronic variants. Furthermore, two patients carried a previously reported variant, c.3270C > T (p.Ser1090Leu). Intronic variants of VWF exon 26 and their pathogenicity as shown in Table 4 among the identified variants, p.Asn1116Ser was detected in two unrelated patients, both with mild bleeding phenotypes. The p.Ser1090Leu variant, previously reported, was found in three patients, consistent with VWD1 diagnostic criteria. These patients were confirmed to have type 1 VWD through phenotypic assessments. Although the clinical significance of most of the detected variants remains to be elucidated, their discovery contributes to a better understanding of the genetic landscape of VWD within the Saudi population.

**Table 1:** Demographic characteristics and laboratory parameters for subjects with an identified variant(s) in VWF exon 26

Member no.	Clinical diagnosis	Family	Gender	Age	Blood group	Bleeding score	Bleeding pattern	VWF:Ag	VWF:RCO	FVIII:C	Nucleotide change (NM_000552)	Protein-level description
1	IC	1	Female	26	O +ve	4	Bruising, gum, tooth extraction	58	39.1	72.9	c.3380-120C > T	No known protein effect (intronic)
2	UAFM	1	Female	53	O +ve	6	Bruising, minor wound, surgery, menorrhagia	178.5	150.1	137.5	c.3380-120C > T	No known protein effect (intronic)
10	IC	3	Female	46	O +ve	7	Surgery, menorrhagia, muscle hematoma	39	27	59	c.3426T > C	p.Phe1142Leu (Missense)
											c.3485C > T	p.Arg1162Ter (Nonsense)
											c.3486A > G	p.Arg1162Arg (Synonymous)
											c.96_3380-91delACATTA	Frameshift variant
											c.3538 + 59A > C	Potential splice site alteration
											c.3538 + 126T > C	Potential splice site alteration
											c.3380-176A > G	No known protein effect (intronic)
											c.3538 + 160A > C	Potential splice site alteration
											c.3538 + 165C > T	Potential splice site alteration
											c.3538 + 179A > G	Potential splice site alteration
											c.3538 + 275A > A	No known protein effect (intronic)
23	AFM	8	Female	32	O +ve	5	Epistaxis, bruising, gum, tooth extraction, menorrhagia, postpartum)	76	57	147	c.3414C > T	p.Phe1138Leu (Missense)
24	UAFM	8	Female	28	O +ve	3	Bruising, menorrhagia	73	83	139	c.3414C > T	p.Phe1138Leu (Missense)
33	UAFM	10	Female	23	O +ve	0	---	75	59	146	c.3414C > T	p.Phe1138Leu (Missense)
38	AFM	12	Female	22	O +ve	2	Bruising, gum	58	50	98	c.3380-74A > A	No known protein effect (intronic)
43	IC	14	Female	19	A +ve	2	Gum, menorrhagia	30	40	51	c.3414C > T	p.Phe1138Leu (Missense)
45	AFM	14	Male	60	O +ve	0	---	46	38	68	c.3414C > T	p.Phe1138Leu (Missense)
46	IC	15	Female	23	O +ve	1	Menorrhagia	54	37	76	c.3414C > T	p.Phe1138Leu (Missense)
47	AFM	15	Female	29	O +ve	0	---	52	38	81	c.3414C > T	p.Phe1138Leu (Missense)
48	UAFM	15	Female	34	O +ve	1	Menorrhagia	92	56	110	c.3414C > T	p.Phe1138Leu (Missense)
50	UAFM	16	Male	8	O +ve	0	---	83	61	120	c.3538 + 142T > C	
58	IC	19	Female	24	A +ve	6	Gum, surgery, menorrhagia	57.3	43	82	c.3414C > T	p.Phe1138Leu (Missense)
60	UAFM	19	Female	54	O −ve	1	Gum, menorrhagia	> 160	49	86	c.3414C > T	p.Phe1138Leu (Missense)
28	IC	---	Female	28	O +ve	7	Bruising, minor wound, Hematuria, GI, gum, menorrhagia, muscle hematoma	51.9	38	84	c.3414C > T	p.Phe1138Leu (Missense)
29	IC	---	Female	31	O +ve	10	Bruising, minor wound, tooth extraction, menorrhagia, postpartum	36	32	61	c.3414C > T	p.Phe1138Leu (Missense)

AFM, Affected family member, Het: Heterozygous; Hom, homozygous; IC, index case; UAFM, unaffected family member.

**Table 2:** Exonic variants of VWF exon 26

	Rs number	Variant type	Nucleotide change (NM_000552)	Codons	Protein change (NP_000543)	No. in VWD cohort (%)	No. in control group	*P* value
1	Rs560397436	Synonymous	c.3414C > T	aaC/aaT	p.Asn1138=	12 (20%)	20 (20%)	0.565
2	Rs535693463	Synonymous	c.3426T > C	tgT/tgC	p.Cys1142=	1 (1.6%)	3 (3%)	
3	Rs566672558	Missense	c.3485C > T	cCa/cTa	p.Pro1162Leu	1 (1.6%)	3 (3%)	
4	Rs546732699	Synonymous	c.3486A > G	ccA/ccG	P.Pro1162=	1 (1.6%)	7 (7%)	

*P* value ≤0.05 is significant.

**Table 3:** Pathogenicity of identified variants in VWF exon 26*

	Rs number	MAF (1000 Genomes global)	Highest population MAF	SIFT	Polyphen	CADD	ClinVar
1	Rs560397436	0.03	0.11	—	—	0.289	VCV000256667 benign, likely benign
2	Rs535693463	0.08	0.35	—	—	2.194	VCV000256668 benign, likely benign
3	Rs566672558	0.08	0.35	Deleterious (0.03)	Probably damaging (0.995)	25.7	VCV000256669 benign, likely benign
4	Rs546732699	0.08	0.35	—	—	1.124	VCV000256670 benign, likely benign

*Extracted from Ensembl (accessed March 2023).

**Table 4:** Intronic variants of VWF exon 26 and their pathogenicity*

	Rs number	Variant type	Nucleotide change (NM_000552)	MAF (1000 Genomes global)*	Highest population MAF*	CADD	Delta score (DS)	No. in VWD cohort (%)
1	rs749182592	del	c.96_3380-91delACATTA	0.08	0.38	0.139	—	1 (1.6%)
2	rs1165370853	SNV	c.3380-74A > A	—	**<0.01**	5.872	9, 35, 9, 1, 0.15, 0, 0, 0	1 (1.6%)
3	rs539367870	SNV	c.3538 + 59A > C	0.006	0.05	0.655	−27, −10, −9, 1, 0, 0, 0, 0	1 (1.6%)
4	rs570696070	SNV	c.3538 + 126T > C	0.076	0.35	0.054	40, 5, −27, −4, 0, 0, 0, 0	1 (1.6%)
5	rs1325694003	SNV	c.3538 + 142T > C	—	**<0.01**	2.675	21, −11, −11, 12, 0, 0, 0, 0	1 (1.6%)
6	rs145909478	SNV	c.3380-176A > G	0.076	0.35	0.908	−1, −20, −19, −8, 0, 0, 0, 0	1 (1.6%)
7	rs575023973	SNV	c.3380-120C > T	—	**<0.01**	0.615	−9, 36, 5, −37, 0.04, 0, 0, 0	2 (3.3)
8	rs183069788	SNV	c.3538 + 160A > C	0.076	0.34	8.595	2, 39, 2, −45, 0, 0, 0, 0	1 (1.6%)
9	rs146955687	SNV	c.3538 + 165C > T	0.076	0.34	0.14	−21, 44, 35, −40, 0, 0, 0, 0	1 (1.6%)
10	rs528134885	SNV	c.3538 + 179A > G	0.075	0.34	1.65	−1, 48, 0, −26, 0, 0, 0, 0	1 (1.6%)
11	rs140221725	SNV	c.3538 + 275A > A	0.076	0.35	2.44	−8, 37, −8, 4, 0, 0, 0, 0	1 (1.6%)

AG, acceptor gain; AL, acceptor loss; DG, donor gain; DL, donor loss; DP, delta positions; DS, delta score.

*Extracted from Ensembl (accessed March 2023).

^Obtained from Ensembl VEP.

## 4 Discussion

VWD is recognized globally as the most prevalent inherited bleeding disorder, resulting from quantitative and/or qualitative defects in VWF, which is encoded by the VWF gene [26]. Type 1 VWD is characterized by a mild presentation associated with reduced VWF levels [27]. The previous study investigated exon 18, while this study focuses on exon 26, with comparisons to the well-characterized exon 28. Previous studies have identified disease-causing variants in the VWF gene in approximately 65% of individuals affected by VWD [28], although this identification is complicated by the heterogeneity of the disease’s phenotypic and genotypic characteristics [29]. Limited data exist on the genotypic characteristics of VWD1 in the Saudi population [30]. Our study included a case group of 22 ICs diagnosed with VWD1 and their first-degree AFM, totaling 60 participants, while the control group consisted of 100 unrelated healthy individuals [31]. Phenotypic characteristics and genotypic analysis of VWF exon 18 have been published previously [32]. Initial findings suggested a unique genotypic profile for the Saudi population compared to others [33]. While exon 28, being the largest exon of VWF, is known for harboring numerous VWD-related variants, only one variant (Rs61749370) was found that potentially explains bleeding symptoms in a single family out of 22 [34]. This current study extends the genotypic analysis to VWF exon 26. Within the VWF exon 26, we identified four exonic and 11 intronic variants in 28% of the VWD1 cohort [35]. All variants were predicted to be benign, except for one missense variant (c.3485C > T) [36]. The benign status of the exonic variants was confirmed by ClinVar, indicating that they are unlikely to cause the disease [37]. Furthermore, these variants did not segregate with the VWD phenotype [38]. All variants are described at the protein level using three-letter amino acid codes (e.g., p.Asn1116Ser). In a parallel analysis involving 100 apparently healthy controls, the identified exonic variants were found at equal or even higher frequencies than in the VWD cohort [39]. Overall, these results reduce the likelihood that any of the identified variants are responsible for VWD1 [40]. However, this does not rule out their potential influence on VWF antigen levels, which could render them disease-associated variants, possibly acting in combination with other genetic or environmental factors [41]. It is well established that certain common VWF variants can affect VWF antigen levels and/or activity, either individually or collectively [42,43]. Although synonymous variants are generally considered to have no structural impact on proteins, some may alter mRNA structure, thereby affecting transcription and translation processes [44]. Furthermore, it is important to recognize that prediction tools and information available in the ClinVar database primarily focus on the effects of individual variants, overlooking the potential interactions of multiple variants that are significant in multifactorial conditions like VWD1 [45]. Variability in VWF antigen levels and activity across different ethnicities has been well documented [46]. Genomic studies of diverse populations indicate that both the exonic and intronic regions of the VWF gene are highly polymorphic, with some variants being specific to certain ethnic groups [47]. For example, variants such as p.Met740Ile, p.His817Gln, and p.Arg2185Gln are rare in European VWD patients but occur frequently in healthy African-American individuals [48]. The presence of such ethnic-specific variants likely accounts for the observed disparities in VWF antigen levels and activity among different populations [49]. The prevalence of VWF variants in exon 26 was compared with findings from other populations, including studies conducted by Krahforst *et al.* in Germany, Veyradier *et al.* in France, Ahmed *et al.* in Pakistan, Yadegari *et al.* in Iran, and Flood *et al.* in the USA [50,51,52,53,54]. Investigating VWD patients from a variety of ethnic backgrounds will enhance our understanding of the roles that both common and rare variants play in the molecular genetics of VWF-related conditions [55]. Interestingly, the frequency of the c.3414C > T variant in our cohort, which was detected in 20% of subjects, is notably higher than the minor allele frequency (MAF) reported in various genomic studies [56], suggesting a potential population-specific enrichment [57]. This observation underscores the importance of considering ethnic variations in VWF genetics when interpreting clinical data and implementing treatment strategies [58]. The rarity of certain intronic variants (c.3380-74A > A, c.3538 + 142T > C, and c.3380-120C > T) that were not found in the 1000 Genomes Project and have a reported MAF of > 0.01 further emphasizes the genetic diversity in the Saudi population and the need for localized genomic studies [59]. Moreover, the phenotypic expression of VWD can vary significantly among individuals, influenced not only by genetic factors but also by environmental aspects such as lifestyle, diet, and overall health status [60]. Future studies should incorporate a comprehensive approach that includes environmental factors alongside genetic analyses to provide a more holistic understanding of VWD manifestations [61]. Additionally, advanced genomic techniques, such as whole exome sequencing and next-generation sequencing, could unveil more complex interactions among variants and their cumulative effects on VWF function [62]. In conclusion, despite the limited number of variants identified in this study, the evidence of differences in allele frequency compared to global reports highlights the impact of ethnic origin on the VWF gene and underscores the genetic diversity within the Saudi population [63]. Establishing genomic studies in conjunction with population databases that include both healthy and affected individuals will facilitate a better understanding of genetic characteristics not only in VWD but also in other multifactorial diseases [64]. Furthermore, collaborative efforts involving multiple research institutions may enhance the discovery of novel variants and contribute to the development of targeted therapies tailored to specific populations [65].

## 5 Conclusion

This study has identified 4 exonic and 11 intronic variants in VWF exon 26; however, none appear to account for the clinical phenotype associated with VWD1. Nevertheless, this does not rule out their potential influence on VWF antigen levels. Future investigations involving sequencing additional exons or employing next-generation sequencing to analyze the entire VWF gene would be beneficial in uncovering potentially pathogenic variants. Our findings indicate a notable enrichment of certain VWF variants within the Saudi population, with a prevalence that exceeds what is recorded in global databases. The genetic disparities observed in comparison to other populations underscore the necessity for a national sequencing initiative in Saudi Arabia. Such a project would enhance our comprehension of the implications of both common and rare variants in the molecular genetics of multifactorial diseases, including VWD1 [66,67,68]. One of the key strengths of this study is its focus on a specific population, which allows for the identification of unique genetic variants that may not be prevalent in other ethnic groups. Additionally, the study’s robust sample size of both AFM and UAFM provides valuable insights into the inheritance patterns of VWD1. However, the study also has limitations. The relatively small number of identified variants may restrict the overall conclusions regarding the genetic basis of VWD1 in the Saudi population. Moreover, the reliance on a single exon for variant analysis may overlook other potentially significant mutations scattered throughout the VWF gene. Future research should aim to incorporate larger sample sizes and more comprehensive genetic analysis to build a clearer picture of the genetic landscape associated with VWD1.
